# Extreme hypernatremic dehydration due to potential sodium intoxication: consequences and management for an infant with diarrhea at an urban intensive care unit in Bangladesh: a case report

**DOI:** 10.1186/s13256-015-0611-y

**Published:** 2015-06-02

**Authors:** Sumon Kumar Das, Farzana Afroze, Tahmeed Ahmed, Abu Syed Golam Faruque, Shafiqul Alam Sarker, Sayeeda Huq, M Munirul Islam, Lubaba Shahrin, Fariha Bushra Matin, Mohammod Jobayer Chisti

**Affiliations:** 1 Centre for Nutrition and Food Security (CNFS), International Centre for Diarrhoeal Disease Research, Bangladesh (icddr,b), 68 Shaheed Tajuddin Ahmed Sarani, Mohakhali, Dhaka 1212 Bangladesh; 2 School of Public Health, Faculty of Medicine and Biomedical Sciences, The University of Queensland, Herston Road, Herston, QLD 4006 Australia; 3 Clinical Service Centre, icddr,b, 68 Shaheed Tajuddin Ahmed Sarani, Mohakhali, Dhaka 1212 Bangladesh

**Keywords:** Bangladesh, Diarrhea, Hypernatremia, Infant, Oral rehydration salt

## Abstract

**Introduction:**

Hypernatremia (serum sodium ≥150mmol/L) is one of the most life-threatening complications of childhood diarrhea, and its management remains challenging, even in a highly advanced critical care setting. This case report describes the acute clinical course and 3-month neurological follow-up after discharge of an infant with extreme hypernatremia in an intensive care unit in Dhaka, Bangladesh.

**Case presentation:**

A 6-month-old Asian Bangladeshi girl of middle-class socioeconomic status was admitted to the intensive care unit of our institution in 2012 with acute watery diarrhea, lethargy and hypernatremia (208mmol/L serum sodium). She had a history of taking excess oral rehydration salt: five packets each, inappropriately prepared, rice-based, properly diluted, glucose-based oral rehydration salt. Her hypernatremia was treated exclusively with oral rehydration salt solution. She experienced seizures on the third day of her hospitalization and was treated with anticonvulsant drugs. Later in the course of her hospitalization, *Enterobacter* spp bacteremia was detected and successfully treated with ciprofloxacin. Although magnetic resonance imaging of her brain at discharge showed cerebral edema, brain magnetic resonance imaging appeared normal at a follow-up examination 3 months after discharge. Electroencephalograms taken at discharge and at her 3-month follow-up examination also appeared normal.

**Conclusions:**

Successful management of extreme hypernatremia with only oral rehydration salt did not result in observable neurological consequences, which emphasizes the importance of the use of oral rehydration salt for the clinical management of childhood hypernatremia.

## Introduction

Serum sodium is a key component of extracellular fluid, which regulates water balance to maintain homeostasis [1]. However, excess serum sodium, or hypernatremia (≥150mmol/L) [1], is often associated with life-threatening neurological deficits caused by the hyperosmolar effect of hypernatremia itself. Inappropriate correction of hypernatremia with a rapid fall of serum sodium is also associated with a high fatality rate [2]. Common life-threatening conditions are hemorrhage, thrombosis, subdural effusion from hypernatremia and cerebral edema caused by a rapid decrease in serum sodium [3]. Improperly prepared oral rehydration salt (ORS) solution, such as preparation with incorrect proportions [4], and intake frequency have often been reported to cause different degrees of severity of hypernatremia [5,6]. Childhood diarrhea caused by rotavirus infection often presents with watery stools, which is associated with the body’s conservation of sodium loss via stools [7,8]. Consumption of appropriately prepared ORS in rotavirus-caused diarrhea may still result in a hyperosmolar condition [9,10]. Children become excessively thirsty, which often results in caregivers’ frequently administering ORS to quench thirst and ultimately leads to severe and extreme hypernatremia [5,6].

Dhaka Hospital is the largest diarrheal disease hospital in Bangladesh. It is the only referral hospital in Bangladesh for diarrheal disease management and related complications, especially electrolyte imbalances. The hospital receives more than 140,000 patients with diarrhea annually, among whom 60% are children under the age of 5 years [11]. Very recently, a child younger than 5 years of age was admitted to the intensive care unit (ICU) of this hospital with diarrhea and 208mmol/L serum sodium. To the best of our knowledge, we report the first case of a child with such an extreme level of serum sodium corrected by simple administration of ORS [12].

## Case presentation

A 6-month-old Asian Bangladeshi girl was brought to our hospital in 2012. She was the third-born child of her non-consanguineous parents and had been delivered normally at home. She was exclusively breast-fed from birth to 2 months of age and then received diluted formula with breast milk. She had received immunizations as per the national Expanded Program on Immunization schedule and did not have any unusual previous illnesses. Her developmental milestones were age-appropriate. Her father was a petty businessman, and her mother was a housewife. The family’s household monthly income was $150 to $200, which is considered middle-class in Bangladesh.

At triage, the initial reported symptoms were acute watery diarrhea with no sign of dehydration; however, as the baby appeared abnormally ill to the attending nurse, she was transferred to the longer-stay ward (LSW). Shortly thereafter (within 2.5 hours), she was relocated to the ICU by the attending physician in the LSW for management of extreme lethargy. In the ICU, her mother reported her child had experienced passage of moderate volumes of watery stool for the previous 2 days, fever for the same duration and lethargy for the past 12 hours. Her bowel movement frequency was 12 to 15 times per day. The stools did not contain visible blood or mucus. Her axillary temperature was 39.4°C. No vomiting, rash or convulsions were observed. For the child’s issues, the mother initially consulted a physician at a nearby community clinic and was advised to provide ORS solution and paracetamol. The parents did not receive any demonstration of appropriate ORS preparation. As the child had received inappropriate treatment, she became restless, extremely thirsty and lethargic, which prompted the mother to bring her child to the Dhaka Hospital of the International Center for Diarrheal Disease Research. Further inquiry of the mother in the ICU revealed that her baby had received excess amount of ORS instead of her routine diet, as the child had excessive thirst. The child received less than one-half of her normal diet, 5 packets of rice-based ORS inappropriately prepared (1 packet of rice powder dissolved and boiled in 500ml of water, instead of 1100ml of water) and 5 packets of properly diluted, glucose-based ORS prior to admission. The child passed urine a few hours prior to admission.

The patient’s physical examination revealed that she was lethargic and irritable upon touch. Her axillary temperature was 39.4°C, her pulse rate was 134 per minute with normal volume, her blood pressure was 100/50mmHg, her capillary refilling time was 3 seconds and her respiratory rate was 54 breaths per minute. She weighed 5.9kg and had a recumbent length of 63cm. According to her anthropometrics, her Z-scores for weight for age, weight for length and length for age were −1.29, −0.40 and −1.21, respectively. She did not present with any pallor or signs of dehydration, edema or cyanosis. Although she was mildly tachypneic, other related clinical signs were normal (breathing, heart sounds), and there was no observable lower chest wall indrawing or adventitious sounds in lungs. Her abdomen was soft, not distended and not tender, and her bowel sounds were active. Her blood glucose was 11.9mmol/L (measured at bedside), and her arterial oxygen saturation was 99% in room air.

On the basis of our patient’s history and physical examination, we determined that her initial problems were acute watery diarrhea, lethargy with excessive thirst and sepsis. On the basis of her lethargy and excessive thirst, our differential diagnoses were hypernatremia and encephalopathy.

The results of our initial investigations are given in Table 1. The chest X-ray, stool routine microscopic examination (RME) and urine RME revealed normal findings. Her blood culture showed growth of *Enterobacter* spp, but a rectal swab culture showed no growth of any organism.

**Table 1:** **Initial assessment at hospital admission (day 1)**

**Indicators**	**Results**
Hemoglobin	9.9g/dl
Total white blood cells	8,900/mm^3^ blood
Neutrophils	44.5%
Lymphocytes	42.0%
Total calcium	1.86mmol/L
Total magnesium	1.22mmol/L
Serum creatinine	47μmol/L

Initially, the child was treated with intravenous (IV) ampicillin and gentamicin for probable sepsis. Within a few hours of admission, her serum electrolyte report became available, which revealed extreme hypernatremia (208mmol/L) and hypocalcemia (1.86mmol/L). Correction of hypernatremic dehydration was achieved by administering ORS via a nasogastric tube, owing to lack of an absolute indication for IV fluid for this child. The required volume was calculated by using the formula [10 ÷ (molecular concentration of sodium in a given solution − measured serum sodium)/(0.6 × weight in kilograms + 1) = liters/24 hours], with the goal of not reducing serum sodium by >10mmol/L over the course of 24 hours (Table 1). Her ongoing serum sodium loss with each purging was also adjusted by ORS administration as required. Her hypocalcemia was corrected by administering a single bolus of IV calcium gluconate (0.5ml/kg after dissolving with the same volume of normal saline) over 10 minutes, followed by oral calcium supplementation. However, her fever remained unresponsive. Her blood culture (sampled on day 1 of admission and confirmed by report on day 3) showed growth of *Enterobacter* spp that were resistant to some antibiotics but sensitive to co-trimoxazole, ciprofloxacin, imipenem and meropenem. Thus, at approximately 96 hours of our patient’s admission, her antimicrobial therapy was changed to IV ciprofloxacin on the basis of clinical unresponsiveness and an antibiogram.

After approximately 24 hours of hospitalization, our patient’s serum sodium level was 174mmol/L and decreasing at a rate of >0.5mmol/L/hr. The correction was readjusted after we recalculated the volume of ORS. The ORS regimen and subsequent serum sodium levels are presented in Table 2. To prevent further decline of her serum sodium level, we added salt to her diet (1g/L). On day 3 (approximately 63 hours after admission), she experienced a generalized tonic-clonic seizure. The seizure was difficult to control and required two doses of IV lorazepam (0.1mg/kg/dose, 10 minutes apart), followed by a loading dose of IV phenobarbitone (20mg/kg) and IV phenytoin (20mg/kg). Simultaneously, maintenance doses of phenobarbitone and phenytoin were administered to prevent further attacks of convulsion. After cessation of convulsion, we reevaluated the patient and found no signs of meningisms. Her fundoscopic examination revealed no signs of cerebral hemorrhage or edema. Around 72 hours after her hospital admission, her serum sodium concentration had returned to a normal level (145.8mmol/L). As she experienced no further convulsions, anticonvulsants were gradually tapered over a period of 7 days, following a seizure-free period of nearly 48 hours, with phenytoin followed by phenobarbitone. Simultaneously, her general condition gradually improved, and her diarrhea resolved by day 6. She became alert and responsive with no apparent neurological deficits, and she was discharged to home on day 12.

**Table 2:** **Patient’s clinical course and laboratory investigations**

	**Day of admission**			
	**Day 1**	**Day 2**	**Day 3**	**Day 4**
Weight (g)	5900	6100	6700	6800
G-ORS^a^ (ml/hr)	14.5	20	25	–
Serum sodium (mmol/L)	208.2	174	160.3	145.8
Serum potassium (mmol/L)	3.95	4.5	4.88	4.83
**Serum chloride (mmol/L)**	**166.2**	**144.1**	**131.3**	**119.8**
**Serum bicarbonate (mmol/L)**	**26.4**	**22.2**	**20.8**	**15.3**
**pH**	**7.46**	**–**	**–**	**–**
Glucose (mmol/L)				
12:00 am	11.9	15.00	–	–
7.00 am	10.3	6.50	4.80	–
12.00 pm	11.3	4.00	–	–

^a^G-ORS, Glucose-based oral rehydration salt.

**Figure 1:**
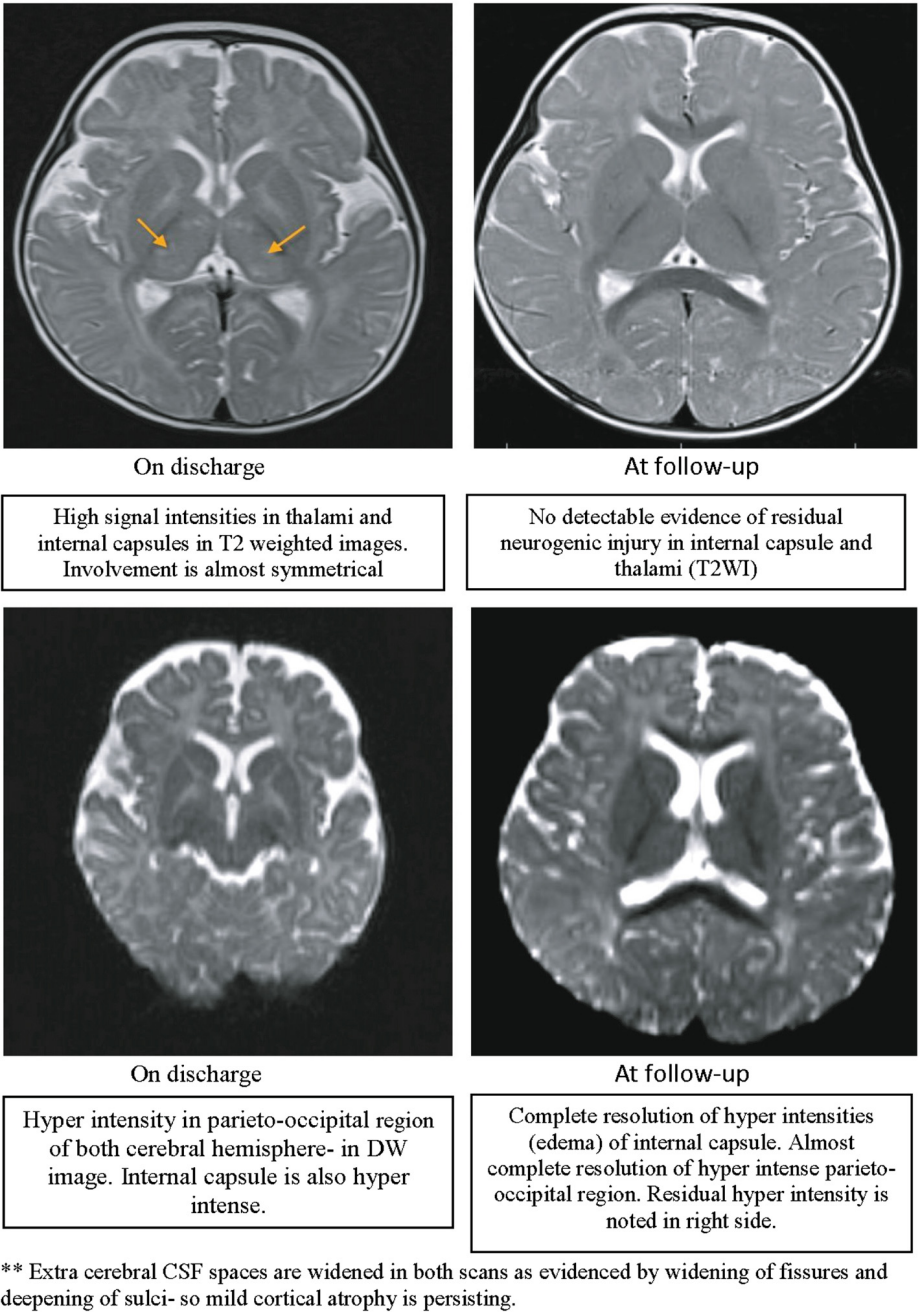
Magnetic resonance imaging scans of the patient’s brain. Images show the acute changes due to hypernatremia at hospital discharge and 3-month follow-up.

To look for any neurological complications associated with hypernatremia, electroencephalography (EEG) and magnetic resonance imaging (MRI) were performed before discharge. The EEG findings revealed a focal slowing that was restricted to the left posterior temporal and occipital regions. MRI showed edema in the internal capsule, thalamus and parieto-occipital cortex of both cerebral hemispheres (Figure 1). Follow-up EEG and MRI (Figure 1) performed 3 months after discharge appeared normal, with complete resolution of edema.

## Discussion

A recent published case report described a patient with hypernatremia with a serum sodium level of 201mmol/L [13], including the patient’s clinical course and management by simple adjustment of ORS solution. Our patient was a child who was admitted to the hospital with a serum sodium level of 208mmol/L, which is the highest level of serum sodium concentration ever reported in Bangladesh. This is the second case involving a patient with serum sodium over 200mmol/L who was successfully managed exclusively with ORS solution [13]. This supports the effectiveness of ORS in correcting hypernatremic dehydration among children [13]. The findings also suggest that hypernatremic dehydration can be treated with ORS following the previous formula, although the ideal decline of serum sodium (0.5mmol/L/hr) might not always be possible [14].

To prevent cerebral edema and convulsion, individuals with hypernatremia should be managed in such a way that the reduction rate of serum sodium occurs at approximately 10 to 12mmol/L/24 hr [15,16]. However, for individuals who develop hypernatremia because of acute salt poisoning rapid correction (1.0mmol/L/hr) within a few hours is also recommended for them to extrude the sodium from the brain to prevent convulsion [14]. As described above, our patient had consumed a large volume of ORS within a very short period and consequently developed hypernatremia acutely. Even after being given careful fluid management for 24 hours, her sodium level was decreased sharply at 34mmol/L. Her development of convulsions on day 3 was difficult to explain. Potential explanations include that the rapid decrease of sodium resulted in cerebral edema, which was confirmed by MRI and EEG at discharge, although fundoscopy showed normal findings during hospitalization. This rapid decline may have contributed to the development of convulsions. Although cerebral edema is ideally managed with 3% NaCl, we could not 0use the hypertonic solution because of a lack of clinical evidence of cerebral edema during our patient’s hospitalization, and therefore her convulsions were managed by the administration of anticonvulsive agents and subsequent supportive dietary management. Thus, performance of neuroimaging during management of seizures may be more helpful. In the case of our patient, a potential cause of her hypernatremia was her consumption of an excess volume of concentrated ORS within a very short period and continuation of only ORS replacing her normal diet to alleviate excessive thirst, which may have contributed to the development of sodium intoxication, resulting in extreme hypernatremia. Disproportionate fecal loss and simultaneous consumption of sodium via the ORS solution led to hyperosmolality in the extracellular space (ECS). The shifting of water from the intracellular space to the ECS resulted in intracellular dehydration and excess thirst [17,18]. Thus, it is important to promote judicious use of ORS in managing hypernatremic dehydration in the absence of an indication for IV correction, such as in patients with severe dehydration or shock, and simultaneously to educate caregivers about the consequences of excess intake and inappropriate preparation of ORS.

There are other potential contributing factors to our patient’s development of extreme hypernatremia. As December is the winter season in Bangladesh, our patient may have had an increased insensible loss of water due to lower humidity. Moreover, she presented with fever, which might have caused excess insensible loss of water. Thus, even with appropriate use of ORS at home during the acute phase management of diarrhea in winter, a patient may develop hypernatremia [9,10], as in the present case.

The initial MRI scan at discharge showed features of cerebral edema potentially resulting from the sharp reduction of serum sodium level within 24 hours, despite careful and very slow correction of dehydration with ORS. However, MRI performed at the 3-month follow-up visit revealed normal findings, which support the fact that adherence to use of ORS solution in managing hypernatremic dehydration helps to ultimately resolve cerebral edema after 3 months of normalization of serum sodium.

## Conclusions

Successful correction of extreme hypernatremia (208mmol/L) with scrupulous adherence to the use of simple ORS solution without any long-term neurological consequences in a young infant may be possible. However, further evaluation in randomized controlled clinical trials on management of hypernatremia with the administration of ORS compared to IV fluid is imperative to accept or refute our observation.

## Consent

Written informed consent was obtained from the patient's mother for publication of this case report and accompanying images. A copy of the written consent is available for review by the Editor-in-Chief of this journal.

## Abbreviations

ECS: Extracellular space
EEG: Electroencephalography
ICU: Intensive care unit
IV: Intravenous
LSW: Longer-stay ward
MRI: Magnetic resonance imaging
ORS: Oral rehydration salt
RME: Routine microscopic examination
